# MTAP loss: a possible therapeutic approach for glioblastoma

**DOI:** 10.1186/s12967-022-03823-8

**Published:** 2022-12-26

**Authors:** C. Pawan K. Patro, Nupur Biswas, Sandeep C. Pingle, Feng Lin, Misa Anekoji, Lawrence D. Jones, Santosh Kesari, Feng Wang, Shashaanka Ashili

**Affiliations:** 1 CureScience, 5820 Oberlin Dr, 202, San Diego, CA 92121 USA; 2 Rhenix Lifesciences, Hyderabad, 500038 India; 3 Department of Translational Neurosciences, Pacific Neuroscience Institute and Saint John’s Cancer Institute at Providence Saint John’s Health Center, CA 90404 Santa Monica, USA; 4 Department of Medical Oncology, Cancer Center, West China Medical School, West China Hospital, Sichuan University, Chengdu, Sichuan China; 5 Present Address: Cancer Science Institute, National University of Singapore, Singapore, 117599 Singapore

**Keywords:** MTAP, Glioblastoma, Cancer, Gene loss, Expression

## Abstract

Glioblastoma is the most lethal form of brain tumor with a recurrence rate of almost 90% and a survival time of only 15 months post-diagnosis. It is a highly heterogeneous, aggressive, and extensively studied tumor. Multiple studies have proposed therapeutic approaches to mitigate or improve the survival for patients with glioblastoma. In this article, we review the loss of the 5′-methylthioadenosine phosphorylase (MTAP) gene as a potential therapeutic approach for treating glioblastoma. MTAP encodes a metabolic enzyme required for the metabolism of polyamines and purines leading to DNA synthesis. Multiple studies have explored the loss of this gene and have shown its relevance as a therapeutic approach to glioblastoma tumor mitigation; however, other studies show that the loss of MTAP does not have a major impact on the course of the disease. This article reviews the contrasting findings of MTAP loss with regard to mitigating the effects of glioblastoma, and also focuses on multiple aspects of MTAP loss in glioblastoma by providing insights into the known findings and some of the unexplored areas of this field where new approaches can be imagined for novel glioblastoma therapeutics.

## Background

Glioblastoma multiforme (GBM) is the most common and lethal form of brain tumors with a median survival time of approximately 15 months after diagnosis [1]. The prognosis for recovery is very low with a recurrence rate of almost 90%. GBM is a complex disease with tumor heterogeneity, so it is essential to find target genes that can be used for patient specific GBM therapeutics as part of a precision medicine approach. There are several strategies to GBM targeted therapeutics [2]. Previous literature has demonstrated the association of specific gene loss such as loss of MTAP (5′-Methylthioadenosine phosphorylase) and GBM therapeutics [3, 4]. One of the strategies includes combining toxic purine analogs such as 2′-fluoroadenine (2FA) with MTAP substrate, MTA. The 2FA + MTA combination has prohibited the tumor growth in MTAP deleted tumors [5].

MTAP, a tumor suppressor gene, encodes a key rate-limiting metabolic enzyme required for the metabolism of polyamines and purines and has a major function in the purine/methionine salvage pathway [6, 7]. MTAP metabolizes 5’-methylthioadenosine (MTA), generated during polyamine biosynthesis, to produce adenine and methionine and salvages them. Homozygous deletion of MTAP is associated with multiple tumors such as mesothelioma, bladder urothelial carcinoma, pancreatic carcinoma, lung carcinoma, leukemia, and glioma among others [5, 8]. Loss of MTAP expression can be caused by the methylation of the MTAP promoter [9]. The deletion of MTAP has multiple implications. Mutation of MTAP results in dysregulated epigenetics and cancer cell stemness [4]. Based on this functional basis, therapeutic strategies have been developed to target MTAP loss for cancer treatment. Firstly, in MTAP-deficient tumor cells, the absence of the salvage pathway sensitizes cells to inhibitors of *de novo* purine synthesis and provides an opportunity to specifically target cancer cells. Secondly, MTAP loss results in the accumulation of MTA and this inhibits the activity of several enzymes, including protein arginine methyltransferase 5 (PRMT5). PRMT5 is a regulatory protein critical for multiple processes such as genome organization, cell cycle regulation, and stem cell differentiation [10]. In addition, dysregulation of PRMT5 is associated with multiple cancers and neurological disorders. Deletion of MTAP results in increased dependency on PRMT5 in cancer cells thus providing the potential avenues for targeted therapies [11]. Besides PRMT5, the vulnerability is also noticed in other upstream and downstream enzymes of PRMT5 including methionine adenosyltransferase II alpha (MAT2A) and Rio domain containing protein (RIOK1) [12].

Although MTAP is functionally relevant in several tumors, MTAP has not been studied extensively and this is evident from the number of published papers in literature databases e.g. PubMed. Keyword search in PubMed for “MTAP” affords only 455 articles to date (PubMed accessed on May 4, 2022, search for “Methylthioadenosine phosphorylase” resulted in 450 articles, and search for “MTAP” and “glioblastoma” resulted in 23 articles. However, the search for “Methylthioadenosine phosphorylase” and “glioblastoma” resulted in only 12 articles of which three were reviews but notably there was no review article specifically on MTAP to date. In this article, we review MTAP with a particular focus on its loss among different tumors and how MTAP loss may be relevant as a therapeutic approach for glioblastoma. In addition, we have also reviewed the relevance of MTAP in other cancers, specific inhibitors, and the associated clinical trials.

### MTAP metabolism pathway

**Fig. 1:**
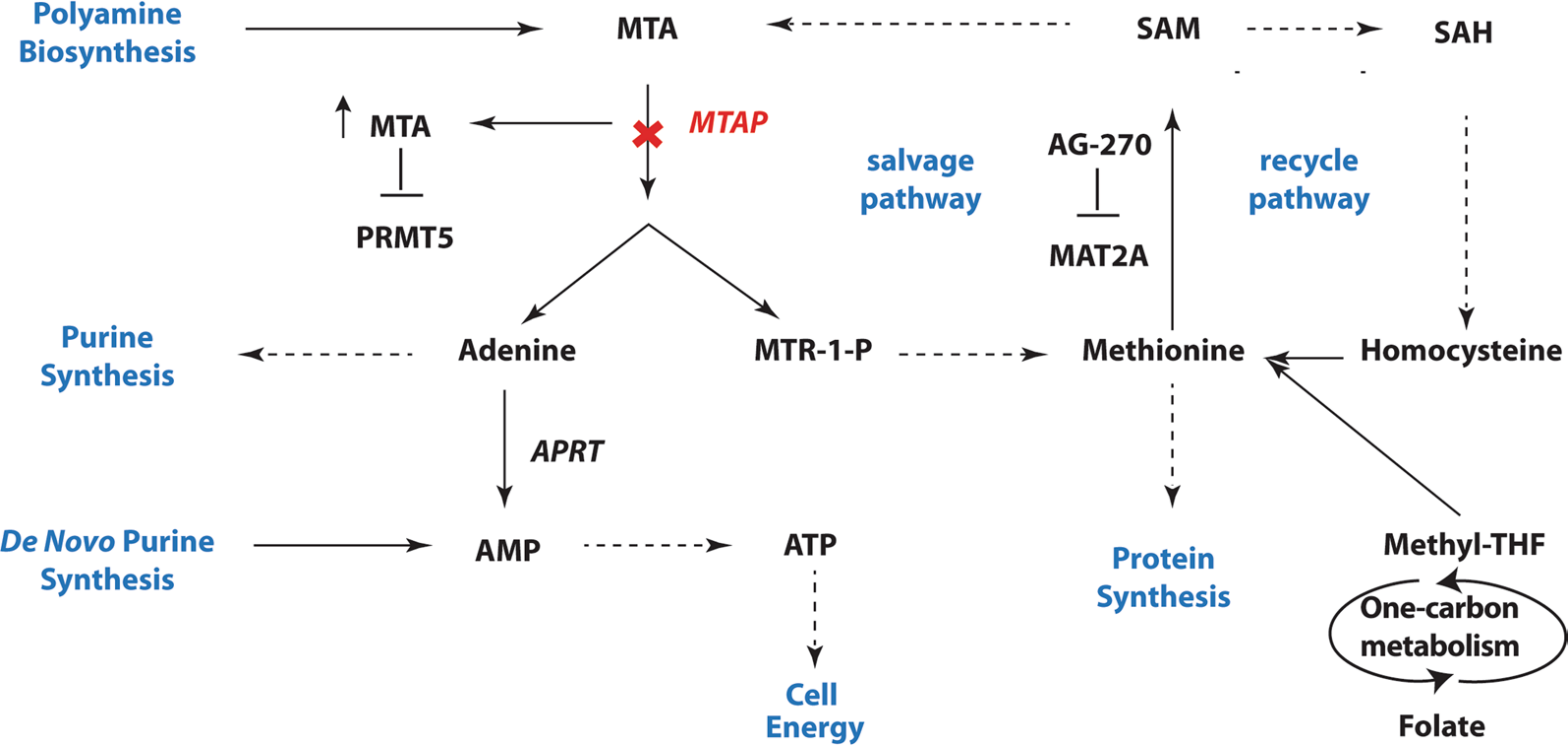
MTAP metabolism pathway. MTAP metabolizes MTA to produce adenine and methionine utilizing a series of intermediate steps. Loss of MTAP (shown by a bold red cross) results in accumulation of MTA and this inhibits PRMT5. Methionine which gets salvaged by MTAP and also can be generated by the folate metabolism pathway is the core of the salvage pathway and the recycle pathway. Methionine gets converted to SAM by MAT2A and SAM can be converted to either MTA or SAH resulting in the salvage and the recycle pathway respectively. AG-270 inhibits MAT2A and this results in lower levels of SAM and thus slows the growth of tumor cells. MTAP is a significant metabolic enzyme because of its involvement in multiple important cellular processes such as protein synthesis, purine synthesis among others as shown in blue. Single step conversions are shown as solid arrows and conversions requiring multiple steps are shown as dashed arrows

In normal cells, MTAP metabolizes 5’-Methylthioadenosine (MTA), a by-product of polyamine biosynthesis, to produce adenine and 5-methylthioribose-1-phosphate (MTR-1-P) [13]. MTR-1-P is converted to methionine by utilizing a series of intermediate steps, and adenine is converted to adenosine 5′-monophosphate (AMP) by utilizing adenine phosphoribosyltransferase (APRT). AMP is also produced by *de novo* purine biosynthesis (Fig. 1). Adenosine triphosphate (ATP) is generated from AMP providing cell energy.

Hence, in MTAP-deficient cells, adenine is not produced from MTA. As a result, for AMP production, cells completely rely on *de novo* purine biosynthesis. Also, cells become more sensitive to inhibitors of *de novo* purine biosynthesis [14, 15] and methionine starvation [9]. These inhibitors include L-alanosine, 6-mercaptopurine, 6-thioguanine (6-TG). This phenomenon is used for selective killing of MTAP deleted cells. The cells are treated with MTA, followed by a high dose of purine analogs like L-alanosine or 6-TG. In normal cells, adenine blocks the conversion of the analog to its toxic nucleotide by phosphoribosylation with 5-phosphoribosyl-1-pyrophosphate (PRPP). However, in MTAP deleted cells, due to lack of adenine, PRPP is present at a high level and converts the analog to its toxic nucleotide, hence killing the tumor cell [16] (Fig. 1).

On the other hand, polyamine biosynthesis connects methionine cycle and one-carbon metabolism [17]. Decarboxylation of s-adenosyl-methionine (SAM) removes one-carbon unit from the methionine cycle. It produces polyamines and MTA. MTA carries the one-carbon unit. With the help of folate derivatives, the one-carbon unit returns back to the methionine cycle for further synthesis of methionine and SAM [18]. Thus MTAP pathway is linked to the folate metabolism pathway through the core component methionine [19, 20] and opens up the possibility of antifolate therapy in treating GBM [21].

### Impact of MTAP loss in GBM

The gene encoding MTAP is located adjacent to cyclin-dependent kinase inhibitor 2A (CDKN2A), cyclin-dependent kinase inhibitor 2B (CDKN2B), and CDKN2B-AS1 in the genomic locus 9p21.3. MTAP loss is associated with the deletion of CDKN2A in the 9p21 locus [22, 23]. MTAP deletion is prevalent in approximately 15% of all solid tumors [20]. In gliomas, deletion of the 9p21 is more frequent in grade IV glioma as compared to grade I glioma [24]. Based on the microarray analysis, Suzuki et al. have shown that MTAP is one of the most frequently deleted genes in glioblastoma [25]. Across 32 TCGA PanCancer Atlas studies, data accessed using cBioPortal [26, 27], MTAP loss is the maximum in GBM based on alteration frequency (deep deletion) and GBM has the maximum alteration frequency for deep deletion (> 40% of the cases); in terms of RNA expression data, GBM has the least median expression for MTAP. Besides GBM, MTAP loss is seen in a wide variety of cancers including mesothelioma, bladder urothelial carcinoma, and pancreatic carcinoma among others, and MTAP is also considered as a tumor suppressor gene [7, 28]. MTAP loss in GBM has its impact on multiple aspects—co-deletion of CDKN2A, prognosis, metabolism pathway, immunosuppressive profile, and cancer cell stemness [4] among others.

### Co-deletion of MTAP and CDKN2A in GBM

MTAP is frequently co-deleted with CDKN2A in a wide variety of cancers such as malignant pleural mesothelioma [29, 30], non-small cell lung cancer [30, 31], and gliomas [32, 33]. Apart from reporting MTAP as most frequently deleted gene, Suzuki et al. have also shown that MTAP and CDKN2A are co-deleted in 15 cases accounting for 50% of the total glioblastoma cases analyzed [25]. GBM, and lower grade glioma (LGG) datasets have shown that MTAP and CDKN2A are co-deleted in more than 40% of the cases in GBM; however, the co-deletion occurs in approximately 8% of the cases in LGG, which include astrocytoma, oligoastrocytoma and oligodendroglioma. GBM had the least expression for both MTAP and CDKN2A compared to LGG. Also, the expression correlation of MTAP and CDKN2A is higher in GBM compared to LGG, being almost twice as highly correlated in GBM.

### Genetic alteration and reduced expression of MTAP in GBM

Homozygous deletion of MTAP is one of the most frequent genetic alterations in GBM. In gliomas, loss of MTAP expression is associated with 9p21 locus deletion and as shown based on the data in cBioPortal it is often co-deleted with CDKN2A in many cancers. Analysis of copy number alteration in the TCGA-GBM dataset by Menezes et al. [7] has shown that there was significantly lower MTAP gene expression in the homozygous deleted group compared to the normal (p < 0.01). Also based on the glioma subtype analysis, they have shown that all the subtypes showed more than 50% MTAP gene expression loss except for the G-CIMP (Glioma-CpG Island Methylator Phenotype) subtype, and the classical subtype showed the highest frequency of loss of MTAP gene expression. It is reported that G-CIMP + subtypes usually co-occur with IDH mutation and have better prognosis [34]. According to the stratified analysis based on different grades of glioma, MTAP expression loss in the high-grade glioma subgroup was almost two-fold greater than in the lower-grade glioma subgroup, although there was no association between MTAP expression and clinicopathological features of patients such as gender and age. Interestingly and contrary to other authors, Menezes et al. have shown that reduced MTAP expression, which is a result of higher MTAP loss frequency in GBM, is associated with better prognosis in adult glioblastoma; although MTAP loss does not affect cell line proliferation, invasion, and migration as shown in their study results based on in vitro models [7]. Menezes et al. further showed that MTAP does not have strong biological importance in gliomas using *in silico* and in vitro models. Moreover, they have shown that MTAP neither has a clinical impact on gliomas nor does it act as a canonic tumor suppressor gene [7]. Based on the TCGA-GBM data analysis, the results of the study have shown that the loss of MTAP expression is due to its loss and not due to its promoter methylation. This is unlike the findings of another study by Hansen et al. [4] that showed an association of MTAP methylation and loss of expression although with a low coefficient of correlation. It is also to be noted that MTAP loss is not observed in some cancers like prostate cancer [18]. Additional studies may be required to explore this aspect in further detail.

### MTAP loss and immunosuppressive profile in GBM

Previous studies [35, 36] have shown that GBM cells utilize multiple strategies to escape the immune surveillance and create an immunosuppressive environment. Hence GBM is characterized by its immunosuppressive nature. MTAP loss results in the accumulation of MTA. The metabolite MTA is associated with cellular context-dependent mechanisms such as targeting the Akt signal pathway, downregulation of TNFα response and host inflammatory response among other associations. Hansen et al. investigated the link between MTAP loss and GBM microenvironment and has shown that loss of MTAP correlates with an immunosuppressive profile in GBM [3]. The results of the study also showed that MTAP loss correlates with differential expression of genes regulating innate or adaptive immune response in experimental cell models and in GBM samples as well. Specifically, MTAP null cells showed lower expression of HLA genes and reduced expression of inflammatory cytokines.

Also, low MTAP expression affects immunosuppressive molecular profiles and has correlations with different immune cells. These correlations manifest in several ways, including alteration in immune cell populations, such as higher M2 macrophages, decreased proportions of γδT cells, and fewer activated CD4 cells which indicates the immunosuppressive context. The study by Hansen et al. concludes with the suggestion that MTAP loss contributes to an immunosuppressive microenvironment in GBM cells. MTAP status should be an important consideration when exploring the immune states and devising immunotherapy-based approaches for GBM treatment.

### MTAP loss promotes cancer cell stemness in GBM

Hansen et al. have shown that a deficiency in MTAP influences the DNA methylome by dysregulation of glioma cell epigenome and promotion of “stemness” in GBM cells [4]. MTAP deficiency leads to increased expression of PROM1/CD133 which results in enhanced tumorigenicity of GBM cells and also promotes glioma stem-like cell (GSC) formation. This is associated with poor prognosis in GBM patients. Although expression of CD133 is associated with poor clinical outcomes in GBM patients and PROM1 expression is required for defining stem cells giving rise to cancer, there are additional markers as well that are identified for different cellular features including cellular characteristics, origins and hierarchy. The results of the study by Hansen et al. support that MTAP loss contributes to the genesis and/or maintenance of the stem-like cancer cells and also regulates specific characteristics of these cells in GBM pathogenesis. These results provide evidence for the role of MTAP loss in GBM pathogenesis, as they have shown that MTAP loss is at the core of the two main components of GBM pathogenesis, which are aberrant DNA methylation, a key feature of cancer cells, and the GBM cell stemness. The findings of this study support and reveal a significant concept of linking aberrant metabolism leading to epigenetic alterations in tumor cells [37, 38]. Since GBM pathogenesis is promoted by MTAP loss, this provides a unique opportunity for GBM therapeutics. Hansen et al. suggest that purine deprivation-based therapy may be a unique approach for the treatment of MTAP-null GBM as they are vulnerable to purine starvation [4].

### MTAP loss in pediatric glioma

Different studies have shown varying results for MTAP loss in pediatric glioma. Based on glioma cell line data, Menezes et al. have shown that there is no loss of MTAP expression in pediatric GBM cell lines, which is in contrast to 50% of cell lines showing loss of MTAP expression in adult glioma cell lines [7]. However, Frazao et al. have shown deletion of MTAP in pediatric gliomas [32]. Frazao et al. also suggested that co-deletion of MTAP and CDKN2A may have therapeutic relevance in pediatric glioma. Further research is required to find additional details and also study the impact of MTAP loss in the context of pediatric glioma. This will facilitate targeted therapy approaches in pediatric glioma as well.

### Relevance of MTAP loss in other cancers

Besides glioblastoma, MTAP loss is also relevant in multiple other cancers as shown across 32 TCGA PanCancer Atlas Studies, data accessed using cBioPortal. Furthermore, MTAP loss is also significant in rare tumors such as chordoma. Chordoma is a rare, aggressive and invasive cancerous tumor of the bone involving the base of the skull, spine and sacrum [39]. Chordoma cases have poor prognosis and are a part of a major group of bone and soft tissue tumors called sarcomas. Previously published studies have shown that MTAP loss has implications on chordoma [28, 40]. Other studies have also shown the association of MTAP with different cancers such as head and neck carcinoma [41], lung cancer [42], prostate [18], colorectal [43], and breast cancer [44]. Even though MTAP is associated with several cancers, the functional relevance and the biological mechanism are yet to be fully understood [45].

### Inhibitors for targeted therapy

There have been only a few inhibitors that are currently undergoing clinical trials in the context of MTAP. In this section, we have reviewed these specific inhibitors, and relevant clinical trials for the drugs and/or inhibitors in the context of MTAP are listed in Table 1.

**Table 1:** Clinical trials in the context of MTAP. Data collected from clinicaltrials.gov on 14th May 2022

Inhibitor / Drug	Condition(s)	Sponsor	ClinicalTrials.gov Identifier	Phase	First Posted (Year)	Recruitment Status
AG-270	Advanced Solid Tumors Lymphoma	Institut de Recherches Internationales Servier	NCT03435250	Phase 1	2018	On-going
IDE397	Solid tumor	Ideaya Biosciences	NCT04794699	Phase 1	2021	On-going
GSK3368715	Neoplasms	GlaxoSmithKline	NCT03666988	Phase 1	2018	Completed
PRT811	Advanced Solid Tumor Recurrent Glioma	Prelude Therapeutics	NCT04089449	Phase 1	2019	Recruiting
AMG 193 Docetaxel	Advanced MTAP-null Solid Tumors	Amgen	NCT05094336	Phase 1 Phase 2	2021	Not started
TNG908	MTAP-deleted solid tumors	NEXT Oncology	NCT05275478	Phase 1	2022	Recruiting
MRTX1719	MTAP deleted solid tumors	Mirati Therapeutics Inc.	NCT05245500	Phase 1| Phase2	2022	Recruiting
Pemetrexed	Chordoma	Saint John’s Cancer Institute	NCT03955042	Phase 1	2019	On-going
Pemetrexed Avelumab	Urothelial cancer MTAP negative	M.D. Anderson Cancer Center	NCT03744793	Phase 2	2018	On-going
Pemetrexed Zimberelimab Etrumadenant	Previously Treated Advanced or Metastatic MTAP Deficient Urothelial Carcinoma	M.D. Anderson Cancer Center	NCT05335941	Phase 2	2022	Not yet recruiting
L-alanosine	Lung Cancer Malignant Mesothelioma Pancreatic Cancer Sarcoma	National Cancer Institute (NCI)	NCT00062283	Phase 2	2003	Completed
L-alanosine	Brain and Central Nervous System Tumors	National Cancer Institute (NCI)	NCT00075894	Phase 1	2004	Completed
Pre-screening study	Advanced Solid Tumors Lymphoma	Agios Pharmaceuticals	NCT03361358	N/A	2017	Completed

Methionine adenosyltransferase II alpha (MAT2A) is expressed in most tissues and cancer cells and is a key enzyme that utilizes methionine to produce S-adenosyl methionine (SAM) in both normal and cancer cells (Fig. 1) [46]. SAM is converted back to MTA completing the cycle (Fig. 1). AG-270 is a safe, tolerable and first-in-class oral MAT2A inhibitor that reduces the proliferation of cancer cells and tumors that lack MTAP [47]. AG-270, currently undergoing a clinical trial (NCT03435250), has been shown to inhibit MAT2A thus blocking the conversion of methionine to SAM and reducing SAM production to a lower level compared to normal. Consequently, the reduction of SAM levels slows down the growth of the cancer cells. The growth of MTAP-deleted cancer cells is blocked as a result of MAT2A inhibition which lowers PRMT5-dependent mRNA splicing and prompts DNA damage [20]. Tumors that have MTAP and p16 co-deletion are sensitive to inhibition of MAT2A and that makes MAT2A an attractive lethal target for MTAP-deleted cancers.

IDE397 is another MAT2A inhibitor that is currently being evaluated in a clinical trial (NCT04794699). IDE397 is a differentiated small molecule inhibitor with great potential for MTAP-deleted cancer patients. IDE397 has demonstrated single-agent anti-tumor activity across various solid tumors including non-small cell lung cancer, gastric and bladder cancer among others.

Besides AG-270 and IDE397 which inhibit MAT2A, in the clinical trial NCT03666988, Fedoriw et al. have shown GSK3368715 as a potential type I PRMT inhibitor [48]. The deficiency of MTAP results in impaired PRMT5 activity and thus sensitizes cancer cells to GSK3368715. To inhibit tumor growth, GSK3368715 works synergistically with PRMT5 inhibitor, GSK3326595. GSK3368715 works by altering exon usage and has strong anti-cancer activity. MTAP loss results in accumulation of PRMT5 inhibitor and this correlates sensitivity to GSK3368715.

Falchook et al. have used PRT811, another PRMT5 inhibitor for treating advanced gliomas. PRT811 is a molecule which worked as a potent, selective brain penetrant PRMT5 inhibitor in animal models of brain tumors [49]. In the clinical trial (NCT04089449), Falchook et al. observed tolerance of PRT811. The inhibition of PRMT5 and anti-tumor activity was observed after multiple doses of PRT811 [50].

AMG 193 is another PRMT5 inhibitor which preferentially binds with MTA-bound state of PRMT5 which is abundant in MTAP-deficient tumors. Clinical trial NCT05094336 uses AMG 193 to check efficacy in advanced MTAP-null solid tumors [51].

TNG908 is also a PRMT5 inhibitor and is being orally administered in the clinical trial NCT05275478 to treat MTAP-deleted advanced solid tumors. TNG908 can bind with PRMT5-MTA complex leading selective inhibition of PRMT5 in MTAP-deleted tumors [52].

MRTX1719 is another inhibitor which targets PRMT5-MTA complex and inhibits PRMT5 activity in animal model of MTAP-deleted tumors [53]. It is being tested in human subjects in the clinical trial NCT05245500.

MTA together with 6-thiogunaine (6-TG) provides selective treatment for cancers with MTAP loss [54]. Therapeutic effects utilizing 6-TG may also be enhanced in MTAP-deficient tumors, when combined with other agents such as methotrexate or pralatrexate. Pemetrexed is an anti-folate drug that inhibits nucleic acid synthesis and disrupts folate-dependent metabolic processes required for cell replication. Pemetrexed also inhibits multiple other enzymes such as thymidylate synthase (TS) and glycinamide ribonucleotide formyl transferase (GARFT) among others in the folate pathway and hence has more clinical significance compared to methotrexate. Several clinical trials are on-going which use Pemetrexed for different types of cancers like chordoma (NCT03955042), and MTAP deficient urothelial cancer (NCT03744793, NCT05335941).

Clinical trials having NCT identifiers NCT00062283 and NCT00075894 use anitibiotic l-alanosine as an inhibitor for MTAP-deficient tumor cells. L-alanosine inhibits purine synthesis by blocking de novo purine synthesis pathway [55]. These trials were initiated in 2003 and 2004, however no results were posted at the time of writing this review.

## Conclusion

MTAP loss has shown potential to create novel GBM therapeutics according to a number of studies; however, Menezes et al. [7] suggest otherwise. Given the lack of clarity regarding the utilization of MTAP deficiency, more studies are warranted to determine the therapeutic potential of MTAP deficiency. This opens new avenues for research in the context of MTAP deficit in cancers including both GBM and lower grade glioma. Palanichamy et al. [56] have shown that methionine and MTA are among differentially regulated metabolites in GBM cells, unlike normal human astrocytes. Also, GBM cells depend on dietary methionine for cell proliferation, colony formation and survival. The results of the study by Palanichamy et al. suggested that the differentially regulated metabolites and their respective pathways serve as potential therapeutic targets for GBM. Interestingly, Barekatain et al. [33] have shown that MTA does not significantly accumulate in vivo because MTA is metabolized by MTAP-expressing stroma; and this leads to metabolic discrepancies in MTA accumulation between in vitro models and primary human tumors. Therefore, this discrepancy must be taken into consideration when precision therapies are being developed for glioblastoma with homozygous MTAP deletion. Moreover, the CDKN2A/MTAP deletion is mostly observed in higher grade glioma [33] and hence tumors at early stage cannot be treated by solely targeting MTAP loss. Any therapeutic approach based on MTAP loss will be applicable to a subgroup of patients. In some rare cases associated with non-small-cell lung cancer and some other cancers MTAP loss occurred in absence of CDKN2A deletion [57]. In such cases targeting MTAP loss may be challenging.

## Data Availability

Not applicable.

## Abbreviations

MTAP: Methylthioadenosine phosphorylase
MTA: Methylthioadenosine
AMP: Adenosine 5′-monophosphate
ATP: Adenosine triphosphate
SAM: S-adenosyl methionine
SAH: S-adenosyl homocysteine
MAT2A: Methionine adenosyltransferase II alpha
APRT: Adenine phosphoribosyltransferase
PRMT5: Protein arginine methyltransferase 5
MTR-1-P: 5-methylthioribose-1-phosphate
Methyl-THF: Methyl tetrahydrofolate
